# Upcycling Tomato Harvest and Processing Residues into Polyphenol-Enriched Cellulosic Films with Tunable Antioxidant and UV-Blocking Properties

**DOI:** 10.3390/foods15061067

**Published:** 2026-03-18

**Authors:** Sarmad Ahmad Qamar, Simona Piccolella, Raffaele Raimondo, Severina Pacifico

**Affiliations:** 1 Department of Environmental, Biological & Pharmaceutical Sciences and Technologies, University of Campania ‘Luigi Vanvitelli’, Via Vivaldi 43, 81100 Caserta, Italy; sarmadahmad.qamar@unicampania.it (S.A.Q.); simona.piccolella@unicampania.it (S.P.); 2 Department of Science and Technology, Parthenope University of Naples, Centro Direzionale, Isola C4, 80143 Naples, Italy; raffaele.raimondo@uniparthenope.it

**Keywords:** tomato cellulose-based bioplastics, tomato polyphenols, cellulose, waste valorization, functional films

## Abstract

The development of bio-based functional materials through the upcycling of agri-food residues represents a sustainable strategy to reduce environmental impact and promote circular economy. This study achieved valorization by combining two tomato by-products: peels exhausted after supercritical fluid extraction and harvest residues mainly composed of stems and field wastes. Polyphenol-rich extract (TPPf) was obtained from peels through ultrasound-assisted maceration and solid-phase extraction, while cellulose from tomato harvest residues (THRs) was converted into carboxymethyl cellulose (THR-CMC, degree of substitution 0.76), as confirmed by structural analyses. Functional bioplastic films were prepared by solvent casting THR-CMC, plasticized with glycerol, and enriched with different TPPf concentrations (0–100 mg/100 mL). Increasing TPPf content enhanced mechanical strength and UV-blocking efficiency, while moderate loading improved moisture barrier properties. The films exhibited notable antioxidant activity (ABTS, DPPH assays) and biodegradability, demonstrating biofunctional performance suitable for food packaging. This integrated valorization strategy highlights the potential of combining agricultural and industrial tomato residues to develop sustainable, biodegradable, and active packaging materials, supporting waste reduction and circular bioeconomy objectives.

## 1. Introduction

The management of agro-food supply chain by-products remains a significant environmental challenge, as most residues are still diverted to low-value uses such as livestock feed, landfilling, or energy recovery by combustion, causing substantial ecological impacts [1]. To promote circularity in industrial biomanufacturing, there is an increasing need for innovative valorization strategies capable of converting agro-food residues into high-value biomaterials and biocomposites [2,3,4].

Among bio-based materials, cellulose has attracted growing attention due to its abundance, renewability, biocompatibility, and biodegradability [5,6,7], finding applications in biomedical scaffolds, food packaging, controlled-release systems, and textiles [8,9,10]. Similarly, polyphenolic compounds recovered from plant residues represent high-value functional additives, providing antioxidant and antimicrobial activities [11,12,13].

In this context, the tomato industry is one of the largest agro-food sectors worldwide and a significant source of organic residues, mainly consisting of tomato harvest residues (THR), including stems and leaves, and peels generated during industrial processing [14]. Although these waste streams are rich in lignocellulosic fibers and bioactive compounds, they remain largely underutilized, representing a unique opportunity for integrated and circular valorization strategies [15,16]. While previous studies have explored cellulose extraction from tomato stems or polyphenol recovery from peels separately [17], the novelty of the present work does not lie in the individual extraction processes, but in the implementation of an integrated and circular multi-stream approach. In this study, tomato harvest residues, representing structural waste, and tomato peels, representing bioactive waste, are simultaneously incorporated into a single functional biomaterial. This integration enabled the creation of a hybrid CMC-polyphenol film with tunable antioxidant and mechanical properties driven by in situ intermolecular interactions. Furthermore, the enhancement of the film through hydrogen bonding and crosslinking between polyphenols and CMC was demonstrated and validated by structural and morphological analyses (Attenuated Total Reflectance—Fourier Transform Infrared Spectroscopy—ATR-FTIR, Scanning Electron Microscopy—SEM). Specifically, cellulose derived from harvest residues was converted into carboxymethyl cellulose (THR-CMC), and both the materials underwent structural and thermal analysis (ATR-FTIR, X-ray Diffraction—XRD, Thermogravimetric Analysis/Derivative Thermogravimetry—TGA/DTG), while the polyphenolic fraction (TPPf) was recovered from exhausted tomato peels through ultrasound-assisted maceration, followed by solid-phase extraction and characterized via Ultra-High Performance Liquid Chromatography—High Resolution Tandem Mass Spectrometry (UHPLC-HR-MS/MS). The combination of THR-CMC and TPPf allowed the development of bioactive, biodegradable, and multifunctional films, characterized in terms of structural, morphological, and functional properties, including physical, mechanical, antioxidant, and biodegradation performance.

Therefore, this study proposes a concrete model of a multi-stream circular economy, transforming agricultural and industrial tomato residues into complementary biopolymeric components, providing an innovative example of integrating agro-food production with sustainable materials, with potential applications in eco-friendly food packaging and food contact materials.

## 2. Materials and Methods

### 2.1. Raw Materials and Chemicals

The chemicals, including sodium hydroxide (NaOH) and acetic acid (CH_3_COOH) were purchased from Carlo Erba Reagents S.A.S. (Milan, Italy). Sodium chlorite (NaClO_2_) and iso-propanol were procured from Merck KGaA (Darmstadt, Germany). DPPH (2,2-diphenyl-1-picrylhydrazyl) and ABTS (2,2’-azino-bis(3-ethylbenzothiazoline-6-sulfonic acid)) were from Sigma Aldrich S.r.l., (Milan, Italy). Tomato harvest residues were obtained from a local farmer of Caserta (Campania region, Southern Italy). The residues were chopped, lyophilized, and crushed into fine powder before further utilization. Tomato peels used in this study were obtained from Della Peruta Vincenzo S.p.A.—Industria Conserve Alimentari (Scafati, Salerno, Italy), a tomato sauce producer. Tomato peels had previously been depleted from the apolar constituents (out of scope of the present investigation) by supercritical fluid extraction (SFE) carried out at Mater S.r.l. (Naples, Italy) and were thus considered an organic agro-industrial by-product from food processing.

### 2.2. Extraction and Characterization of Tomato Polyphenolics

Tomato peel residues after supercritical fluid extraction (SFE) were subjected to ultrasound-assisted extraction (UAE) using ethanol as solvent. Extractions were performed with a solvent-to-matrix ratio of 20:1 (*v:w*) at 20 °C for 30 min per cycle, using a Branson M3800-E ultrasonic bath (Danbury, CT, USA). Three consecutive cycles were carried out, yielding an overall crude extract recovery of 39.7%. The crude extract was purified by solid-phase extraction (SPE) using an RP-8 cartridge, resulting in a final polyphenolic extract yield of 4.6%.

The purified extract was characterized spectroscopically. ATR FTIR spectra were collected using FT-IR (IRXross, Shimadzu, Tokyo-Japan) over the range 4000–500 cm^−1^, with 45 scans and a resolution of 4 cm^−1^ using LabSolutions IR (v1.60). Spectra were visualized using OriginPro 2015 (OriginLab, Northampton, MA, USA). UV-Vis absorption spectra were recorded from 200–800 nm using a spectrophotometer (Cary 100, Agilent, Milan-Italy). UHPLC-HRMS analysis was performed for the profiling of untargeted metabolites (NEXERA UHPLC system, Shimadzu, Tokyo, Japan) coupled with AB SCIEX Triple TOF^®^ 4600 mass spectrometer (AB Sciex, Concord, ON, Canada), equipped with a C18 column (Luna^®^ Omega, 50 × 2.1 mm, 1.6 µm; Phenomenex, Torrance, CA, USA). The mobile phase consisted of water (A) and acetonitrile (B), both containing 0.1% formic acid. The chromatographic gradient was as follows: 5% B from 0.01 to 1.00 min; linear increase to 35% B at 15.00 min; ramp to 95% B at 16.00 min; return to 5% B at 16.01 min, and re-equilibration until 18.00 min. The flow rate was 0.5 mL/min, and the injection volume was 2 µL. Mass spectrometric detection was performed in negative electrospray ionization mode under Information Dependent Acquisition (IDA). Data acquisition combined a TOF-MS survey scan (100–1000 Da, 250 ms) with eight IDA MS/MS scans (80–900 Da, 100 ms each). ESI source parameters were curtain gas (CUR) 35 psi, nebulizer gas (GS1) 60 psi, heated gas (GS2) 60 psi, ion spray voltage (ISVF) −4.5 kV, interface temperature 600 °C, declustering potential −80 V, collision energy 40 V with CES 25. Instrument control and data acquisition were performed with Analyst^®^ TF 1.7, and data processing with PeakView^®^ 2.2.

### 2.3. Recovery and Carboxymethylation of Cellulose from Tomato Harvest Residues

To extract cellulose from tomato harvest residues, alkaline delignification with 5% NaOH at 80 °C for 4 h, followed by washing to neutrality and bleaching with 1.4% NaClO_2_ (pH 3.5–4.0) at 75 °C for 3 h [18]. The purified cellulose was then washed and lyophilized. Carboxymethylation (CMC) was then carried out [19]. Dried cellulose (5 g) was alkalized with 50% NaOH in 2-propanol, then reacted with mono-chloroacetic acid at 50 °C for 4 h. After neutralization with diluted acetic acid, the resulting CMC was thoroughly washed (70% EtOH) and lyophilized. The degree of substitution (DS) of tomato CMC was determined by a titration method, as reported in our previous study [4].

### 2.4. Development of Bio-Based Active Films

Film-forming dispersions were prepared by dissolving THR-CMC (2 g/100 mL) in distilled water under mild stirring at 25 °C. After complete dissolution, glycerol (0.8 g/100 mL) was added as a plasticizer, and the solution was heated at 50 °C for 20–30 min. Different concentrations of tomato polyphenolic fraction (0, 40, 60, and 100 mg/100 mL) were incorporated to enhance the bioactive properties of the films. The mixtures were centrifuged at 3000 rpm to remove undissolved particles, poured into *Petri* dishes, and dried at 40 °C for 24 h. After drying, the films were conditioned and stored in a desiccator at 25 °C and 50% relative humidity until further analysis. The resulting films were designated as F0 (control), F1 (40 mg/100 mL), F2 (60 mg/100 mL), and F3 (100 mg/100 mL), according to their polyphenolic content.

### 2.5. Structural Characterization

The presence of major functional groups in THR-cellulose, THR-CMC, and the developed bioplastic films was evaluated using attenuated total reflectance-Fourier transform infrared (ATR-FTIR) spectroscopy (IRXross, Shimadzu, Tokyo, Japan). Spectra were recorded in the range of 4000–500 cm^−1^ by directly placing dried samples on the ATR crystal. Structural order and hydrogen bonding were further assessed using ATR-FTIR-derived indices [20]. The total crystallinity index (TCI) was calculated as the absorbance ratio at 1370 cm^−1^ to 2900 cm^−1^, representing overall crystalline content. The lateral order index (LOI), indicative of cellulose chain ordering, was determined from the absorbance ratio at 1430 cm^−1^ and 893 cm^−1^. The hydrogen bond intensity (HBI), reflecting the extent of intra- and intermolecular hydrogen bonding, was estimated from the absorbance ratio of the O–H stretching region (~3350 cm^−1^) to the CH_2_ bending vibration (~1315 cm^−1^).

Crystallinity of cellulose and CMC was further examined using X-ray diffraction (XRD) analysis with a diffractometer (GNR Analytical Instrument, Novara, Italy) equipped with a θ/θ goniometer. Measurements were performed using Cu Kα radiation (λ = 1.5406 Å) over a 2θ range of 2–60° with a step size of 0.02°/min. The crystallinity index was calculated using the Segal method [21].

Thermal degradation behavior of THR-cellulose and THR-CMC was analyzed using a thermal analyzer (TGA 4000, PerkinElmer, Milan, Italy). Samples (20 mg) were heated from 30 °C to 600 °C at a heating rate of 10 °C/min under a nitrogen atmosphere (flow rate: 20 mL/min). All ATR-FTIR, XRD, and TGA/DTG spectra were processed and plotted using OriginPro 2025 (OriginLab Corp., Northampton, MA, USA).

Surface morphology of the developed bioplastic films was examined using scanning electron microscopy (SEM) (Carl Zeiss EVO 50-XPV, Oberkochen, Germany). High-resolution images of film surfaces and cross-sections were captured at various magnifications to evaluate surface characteristics and internal structure.

### 2.6. Determination of Physical Properties of Films

The transparency index (TI) of the prepared films was determined by measuring light transmittance at 600 nm (T_600_) using a UV-Vis spectrophotometer (Cary 100, Agilent Technologies, Milan, Italy). TI was calculated as *T*_600_ divided by film thickness, with higher TI values indicating greater optical clarity.

Film thickness was measured using a digital micrometer. Color parameters were determined using a Chroma Meter CR-5 colorimeter (Konica Minolta, Tokyo, Japan). The CIELab color coordinates, L* (lightness), a* (red–green), and b* (yellow–blue), were recorded and used to calculate the whiteness and yellowness indices of the films [7].

Moisture content and water vapor transmission rate (WVTR) were evaluated following previously established methods [4,22]. Briefly, impermeable glass vials containing silica gel desiccant were tightly sealed with the prepared films and placed inside a desiccator containing distilled water. The vials were weighed after 24 h (WVTR_24_) and 48 h (WVTR_48_) to determine water vapor transmission.

### 2.7. Determination of Mechanical Properties of Films

Mechanical properties, including tensile strength, elongation at break, and Young’s modulus, were measured using a universal testing machine (EZ-SX, Shimadzu, Kyoto, Japan). Film specimens were cut into dog-bone shapes (5 cm length, 2 cm width at the ends, and 1 cm width at the narrow center) in accordance with ASTM D638 standards [23] and mounted carefully to avoid misalignment. Tests were conducted at a crosshead speed of 5 mm/min using a 100 N load cell. All measurements were performed in triplicate, and mechanical parameters were calculated as previously described [4].

### 2.8. Functional Properties of Films

#### 2.8.1. UV-Blocking Properties

The UV-blocking capacity of the developed films was evaluated by measuring optical transmittance in the wavelength range of 200–800 nm using a UV–Vis spectrophotometer (Cary 100, Agilent Technologies, Milan, Italy). Film samples were cut into rectangular strips (0.5 × 3.0 cm, width × length) and carefully placed in the sample holder. Transmittance spectra were recorded in both the ultraviolet (200–400 nm) and visible (400–800 nm) regions to assess UV-shielding performance and optical transparency [21]. The percentage of UV-blocking (% UV-blocking) was calculated from the transmittance values according to the equation: % UV-blocking = (1 − *T*/*T*_0_) × 100, where *T* is the transmittance of the sample film and *T*_0_ is the transmittance of the control film (F0) at the same wavelength. For each sample, % UV-blocking values were averaged separately for the UV-B (280–320 nm) and UV-A (320–400 nm) regions. All measurements were performed in triplicate, and results are reported as mean ± standard deviation (SD).

#### 2.8.2. Antioxidant Properties

The antioxidant activity of the films was evaluated using ABTS and DPPH radical scavenging assays [22]. Circular film samples (5 mm diameter) were placed in 96-well microplates, and Trolox (2–32 µM) was used as a positive control. The ABTS^●+^ working solution was prepared by reacting 7 mM ABTS with 2.45 mM potassium persulfate for 12 h in the dark and subsequently diluted to obtain an absorbance value ≤0.7 at 734 nm. For the assay, 300 µL of the ABTS reagent was added to each well containing the film sample, and the decrease in absorbance was measured at 734 nm after incubation. Similarly, for the DPPH assay, 300 µL of a 1.0 × 10^−4^ M methanolic DPPH solution was added to each well, and absorbance was measured at 515 nm after incubation. In both assays, antioxidant activity was expressed as the percentage of radical scavenging, calculated based on the reduction in absorbance of the radical solution in the presence of the film samples compared to the control.

### 2.9. Biodesintegration Assay of THR-CMC-Based Films

The biodesintegration of the films was evaluated using a soil burial test in accordance with the ASTM D6400 standard [7,22,24]. Two specimens (2 cm × 2 cm) from each film were cut and weighed to record the initial weight. The samples were then buried in garden soil contained in small pots with perforated bottoms to enhance oxygen diffusion and microbial activity. The test was carried out at ambient temperature (25 °C) for a total period of 24 days. Film samples were recovered at regular intervals of four days, gently cleaned with tissue paper to remove soil residues, and weighed again to determine weight loss. The biodegradability of the films was expressed as the percentage of mass loss over time, calculated by comparing the initial and final weights of the samples.

### 2.10. Statistical Analysis

All experimental data are presented as mean ± standard deviation (SD) of three independent replicates. Statistical differences among samples were analyzed using one-way analysis of variance (ANOVA), followed by Tukey’s multiple comparison test at a 95% confidence level (*p* < 0.05). Statistical analyses were performed using GraphPad Prism 9 (GraphPad Software, La Jolla, CA, USA).

## 3. Results and Discussion

The workflow of the present study was designed to integrate the valorization of two complementary tomato processing residues into functional biopolymeric films. Initially, the tomato polyphenolic fraction (TPPf) was obtained from exhausted tomato peels after supercritical carotenoid extraction. A thorough chemical characterization of this fraction was performed, since understanding the exact composition of polyphenols is essential to interpret their contribution to the bioactivity, UV-blocking capacity, and mechanical behavior of the resulting films. Following the polyphenol recovery, cellulose was extracted from tomato harvest residues (THR) and subsequently functionalized into carboxymethyl cellulose (THR-CMC). Although the preparation of THR-CMC and TPPf involves energy-intensive steps such as lyophilization and chemical treatments for delignification, these processes were necessary to ensure efficient recovery of high-purity functional components. From a circular economy perspective, the approach aims to maximize the valorization of agro-industrial residues; however, future optimization should focus on reducing the environmental footprint of these processing steps, particularly in view of potential scale-up. This stepwise approach ensured that both structural (cellulose-based) and functional (polyphenol-based) components were fully characterized before integration into hybrid films, allowing for a detailed mechanistic understanding of their interactions and the properties of the final biomaterials.

### 3.1. Characterization of Tomato Polyphenolic Fraction

**Figure 1:**
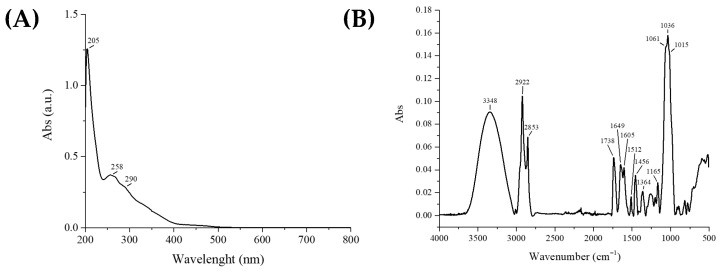
Characterization of the tomato polyphenolic fraction (TPPf) obtained from exhausted tomato peels. (**A**) UV-Vis absorption spectrum; (**B**) ATR-FTIR spectrum.

Tomato peels, a by-product of industrial processing, were initially subjected to supercritical fluid extraction in order to recover the carotenoid fraction. Once this component was depleted, the remaining biomass was further treated using ultrasound-assisted extraction (UAE), with ethanol as solvent. The resulting extract, TPPf, was preliminarily characterized by UV-Vis and ATR FT-IR spectroscopy (Figure 1). The UV-Vis spectrum showed, in addition to a strong peak at 205 nm, bands at 258 nm and a shoulder at 290 nm. These features can be attributed to π→π* and n→ π* transitions typical of conjugated aromatic systems found in polyphenols, flavonoids, and related secondary metabolites (Figure 1A).

The exact nature of these compounds was further elucidated by UHPLC-HRMS analysis, as discussed below. The ATR FT-IR spectrum exhibited intense signals at the following wavenumbers: 3348 cm^−1^, corresponding to O–H stretching vibrations, indicative of hydroxyl groups (phenols, alcohols); 2922 and 2853 cm^−1^, associated with aliphatic C–H stretching (CH_2_, CH_3_); 1738 cm^−1^, related to C=O stretching of esters or carboxylic acids; 1649 cm^−1^, which may be attributed to either aromatic C=C or amide C=O stretching, and 1605 cm^−1^, potentially associated with aromatic C=C or amide N–H bending. These two bands are suggestive of the possible presence of amide-containing compounds, such as phenolamides, which were later confirmed through UHPLC-HRMS analysis. Additional signals at 1456 cm^−1^ (CH_2_/CH_3_ deformation) and at 1165 and 1036 cm^−1^ (C–O stretching of alcohols, esters, and polysaccharides) further support the matrix complexity (Figure 1B).

TPPf was analyzed by UHPLC-HRMS (Table 1), revealing 34 compounds classified as flavonoids (41%), hydroxycinnamic acid derivatives (35%), phenolamides (1%), aromatic glycosides (11%), and jasmonic acid derivatives (12%).

Among the identified metabolites, rutin represented the most abundant compound, accounting for approximately 27% of the total flavonoid content. This finding is consistent with previous reports identifying rutin as the predominant flavonoid in tomato fruits [25]. All compounds were identified through MS/MS spectral interpretation and comparison with authentic standards, where available. Within the flavonoid class, quercetin glycosides were particularly represented (Figure S1). A compound with a deprotonated molecular ion at *m*/*z* 741.1714 was identified as quercetin pentosyl deoxyhexosyl hexoside. Its fragmentation pattern, yielding product ions at *m*/*z* 301.0331 and 300.0260, was consistent with a quercetin glycosylated at position 3, based on the relative abundance of the aglycone and radical ions. According to the literature, this compound corresponds to quercetin 3-*O*-(2’’-*O*-β-apiofuranosyl-6’’-*O*-α-rhamnopyranosyl-β-glucopyranoside), previously reported in tomato peel [26]. The *p*-coumaroyl derivative of the previous compound was also detected (Figure S1A). In fact, the deprotonated molecular ion at *m*/*z* 887.2208 gave the TOF-MS/MS ion at *m*/*z* 741.1851 following the neutral loss of 146.03 Da, corresponding to dehydrated *p*-coumaric acid. Another triglycosylated quercetin derivative, with a deprotonated molecular ion at *m*/*z* 771.2002, was also identified. Upon MS/MS fragmentation, this compound exhibited the sequential loss of a hexose residue (162.05 Da) to form ions at *m*/*z* 601.1463, *m*/*z* 462.0806, and *m*/*z* 301.0348, suggesting a quercetin triglycoside structure. The data support the presence of a hexose substitution at position 7 and a deoxyhexosylhexosyl moiety linked at position 3 of the aglycone. The pentosyl deoxyhexosyl hexosyl derivative of kaempferol (at *m*/*z* 725.1910) was also observed (Figure S1B). The loss of the trisaccharide component led to the aglycone ion at *m*/*z* 285.0384 and to the more abundant radical aglycone at *m*/*z* 284.0311. Naringenin and its glycosides were also identified (Figure S2). Naringenin chalcone ([M-H]^−^ at *m*/*z* 271.0599), the biosynthetic precursor of naringenin, was detected in lower abundance compared with literature values for raw tomato tissues [27]. This reduction is likely associated with its partial transfer to the tomato sauce matrix during industrial processing and extraction, where the chalcone open-ring structure facilitates solubilization in the aqueous phase. Phloretin 3’,5’-di-*C*-glucoside, which was previously isolated from tomato [26], was also tentatively identified. The deprotonated molecular ion exhibited characteristic fragmentation behavior typical of *C*-glycosyl flavonoids (Figure S3). The base peak at *m*/*z* 357.0959 resulted from the sequential neutral loss of two 120.04 Da units, corresponding to cross-ring cleavages of both sugar moieties. A secondary fragment ion at *m*/*z* 477.1381 arose from a single cross-ring cleavage (−120.04 Da). Additionally, the ion at *m*/*z* 387.1065, corresponding to a neutral loss of 90.03 Da from *m*/*z* 477.1381, represents another typical sugar cross-ring fragmentation. Hydroxycinnamoyl-based compounds accounted for approximately 35% of the total phenolic content (Figures S4 and S5). This group was dominated by chlorogenic acids, including mono-, di-, and tri-caffeoyl derivatives of quinic acid (Figure S4). Compounds with deprotonated molecular ions at *m*/*z* 353.0878, 515.1191, and 677.1503 were respectively assigned to 3-*O*-caffeoylquinic acid, 3,5-di-*O*-caffeoylquinic acid, and 3,4,5-tri-*O*-caffeoylquinic acid, based on their accurate masses and MS/MS fragmentation patterns. The three positional isomers of monocaffeoylquinic acid, 3-*O*-, 4-*O*-, and 5-*O*-caffeoylquinic acids, were distinguished by their diagnostic fragment ions. The 5-*O*-caffeoylquinic acid produced a dominant ion at *m*/*z* 191.0550 (quinic acid [M-H-caffeoyl]^−^), while the 3-*O*- and 4-*O*- isomers showed additional fragments at *m*/*z* 179.0344 and 173.0450 (abundant for the 4-O- isomer), corresponding to the loss of water and CO_2_, respectively [28]. In addition to chlorogenic acids, caffeoyl glycosides and their dihydro-derivatives were also detected (Figure S5). Among these, monohexosylated and dihexosylated derivatives of caffeic acid were included [29]. Although present in minor amounts (approximately 1%), the phenolamide *p*-coumaroyltyramine hexoside was detected. The loss of 162.05 Da from the ion at *m*/*z* 444.1656 produced the fragment at *m*/*z* 282.1159, while additional ions at *m*/*z* 162.0452 and 119.0499 were attributed to the *p*-coumaroyl unit (Figure S6). Phenolamides are known to participate in plant defense and stress responses and have been previously described in tomato. Three aromatic glycosides were also identified in the TPPf (Figure S7), in agreement with previous findings reporting similar compounds in ripe cherry tomato fruits [30].

Finally, the jasmonic acid derivatives accounted for about 12% of the total phenolic profile. Two isomeric forms of tuberonic acid hexoside were detected in the extract (Figure S8). A diagnostic difference was observed in the MS/MS fragmentation pattern: while one isomer exhibited both the characteristic aglycone ion at *m*/*z* 207.1017 and the fragment ions corresponding to the sugar moiety, the other isomer lacked the tuberonate ion and showed only the fragment at *m*/*z* 163.114, indicative of the saccharidic portion. Such a difference can be explained by variations in the position or nature of the glycosidic linkage (e.g., *O*-glycosylation vs. ester linkage). Furthermore, the caffeoyl derivative of the previously described compounds was also detected, exhibiting a deprotonated molecular ion at *m*/*z* 549.1868. This latter underwent the neutral loss of a caffeoyl moiety to achieve the ion at *m*/*z* 387.1603. Tuberonic acid, which is a hydroxylated form of jasmonic acid, plays a crucial role in signaling pathways related to stress tolerance, senescence, and plant-microbe interactions [31].

### 3.2. Cellulose Recovery and Functionalization to CMC

**Figure 2:**
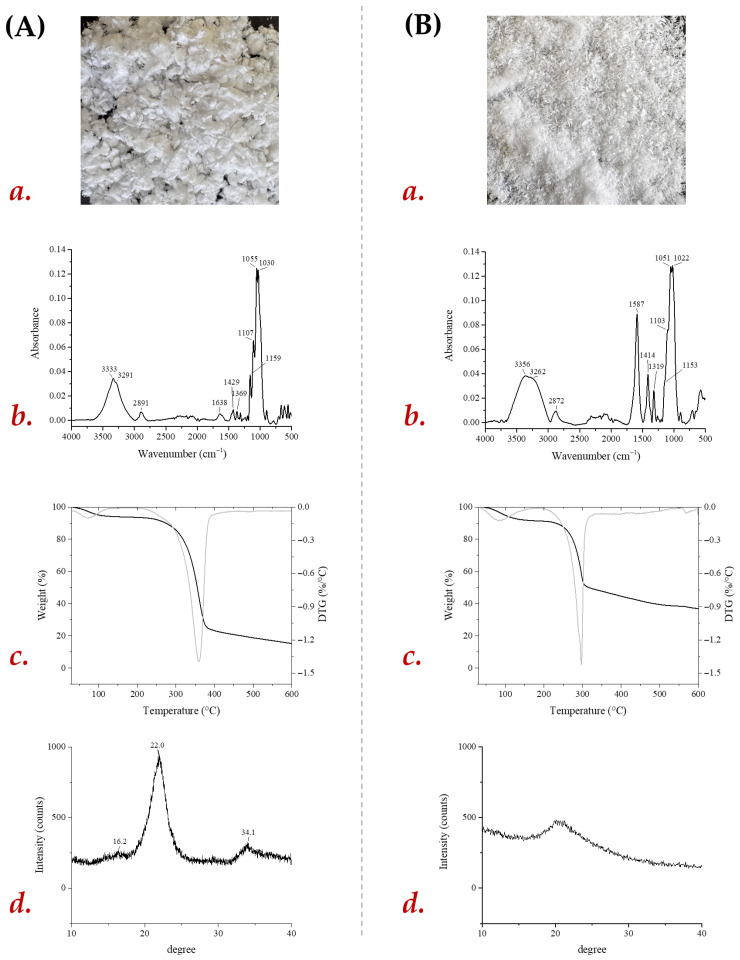
Characterization of (**A**) THR-cellulose obtained from tomato field residues and (**B**) its derived THR-CMC. (**a**). Visual morphology of the dried powders; (**b**). ATR-FTIR spectra; (**c**). thermogravimetric (TGA) and derivative (DTG) curves; (**d**). X-ray diffraction (XRD) patterns.

The cellulose extraction was performed using a two-step delignification: (i) 5% (*w/v*) NaOH(aq) and (ii) 1.4% (*w/v*) NaClO_3_ (pH 3–4), resulting 22% (dry weight) of clear-white crystalline cellulose (Figure 2(Aa)). The alkaline treatment disrupted the carbohydrate-lignin matrix, while NaClO_3_ oxidatively removed lignin by breaking aromatic rings [32]. Although the yield was slightly lower than that reported for other agri-food residues such as sweet sorghum stalk (34%), corn stover (23%), rice husk, and sago seed shells (35%) [33,34,35], THR still represents a valuable and abundant source of cellulose for sustainable material production. Aiming to improve dissolution kinetics and film-forming abilities, the cellulose was functionalized to carboxymethyl cellulose (THR-CMC; Figure 2(Ba)) with a %yield of 0.974 g/g and 0.76 degree of substitution (DS). A DS value above 0.4 suggests complete solubility in water while below 0.4 means that CMC is swellable but insoluble in water [36]. The typical DS of CMC obtained from cellulose via alkalization and carboxymethylation reactions range from 0.4–0.9 [4]. The DS value is pivotal in determining structural/functional properties of bioplastics as higher the DS value reduced the crystallinity of CMC, resulting in increased flexibility, transparency, and elongation at break, making them important for packaging application where high optical clarity is required [3,37].

### 3.3. Structural Characterization of THR-Cellulose and Derived CMC

ATR-FTIR, TGA, and XRD analyses revealed significant structural, thermal, and crystallinity changes between THR-cellulose and THR-CMC. The ATR-FTIR spectrum of THR-cellulose showed broad O–H stretching around 3335 cm^−1^, C–H stretching near 2890 cm^−1^, a minor C=O band at 1734 cm^−1^, CH_2_ rocking at 1312 cm^−1^, and C–O–C vibrations at 1159 cm^−1^. Following carboxymethylation, THR-CMC retained the O–H and C–H bands, albeit slightly shifted (3356, 3262, 2872 cm^−1^), indicating modified hydrogen bonding and partial substitution. New absorption peaks at 1587 and 1414 cm^−1^ confirmed the introduction of carboxymethyl groups, while a minor shift in CH_2_ scissoring (1312 → 1319 cm^−1^) reflected local structural changes [38]. Bands at 895 cm^−1^ and 664 cm^−1^, corresponding to β-glycosidic linkages and O–H bending, were retained, indicating preservation of the polysaccharide backbone [39,40].

TGA analysis showed that THR-cellulose underwent initial moisture loss below 100 °C, corresponding to adsorbed water and residual solvent, followed by sharp degradation between 300 and 400 °C, corresponding to cellulose depolymerization, leaving ~20% residue at 600 °C [41,42,43]. In contrast, THR-CMC exhibited an earlier and broader thermal degradation, with *T*_onset_ around 220–240 °C and *T*_max_ at 295–305 °C, reflecting reduced thermal stability due to carboxymethyl substitution. The introduction of –CH_2_COO^−^ groups disrupts crystalline domains and weakens intermolecular hydrogen bonding, generating thermally labile sites that promote earlier decomposition. DTG profiles further highlighted these differences: THR-cellulose displayed a sharp, well-defined peak at ~360 °C, whereas THR-CMC showed a broader, less intense peak, indicating a more complex degradation pathway involving both the polysaccharide backbone and substituted side chains. THR-CMC also retained slightly higher residual mass (22–24%) compared to cellulose (18–20%), likely due to thermally stable sodium-containing residues.

XRD patterns revealed distinct peaks for THR-cellulose at 22.5° and 15–16° (2θ), corresponding to the (200) and (110)/($\bar{110}$) planes of cellulose I, indicating high crystallinity [44,45]. THR-CMC, in contrast, displayed a broad, less intense peak centered at 20.1° (2θ), indicative of disrupted crystalline domains due to carboxymethylation. Crystallinity, calculated using the Segal method, confirmed this structural change: THR-cellulose had a CrI of 62%, whereas THR-CMC dropped to 24%, consistent with its predominantly amorphous nature. This reduced crystallinity provides a flexible matrix that allows low concentrations of TPPf to interact with cellulose chains via hydrogen bonding, improving water barrier properties. At higher TPPf loadings, the extract tends to form hydrogen-bonded aggregates within the amorphous matrix, which increases mechanical stiffness, while the hygroscopic nature of the polyphenols contributes to higher water vapor transmission. These findings were corroborated by ATR-FTIR-derived structural indices. The Total Crystallinity Index (TCI) slightly decreased from 1.147 in THR-cellulose to 1.134 in THR-CMC, confirming the loss of ordered crystalline domains. In contrast, the Lateral Order Index (LOI) increased from 1.022 to 1.250, reflecting a rearrangement of cellulose chains and local structural order. Meanwhile, the Hydrogen Bond Intensity (HBI) decreased from 4.714 to 1.596, reflecting the disruption of inter- and intra-molecular hydrogen bonding caused by the substitution of hydroxyl groups with carboxymethyl moieties. This reduction aligns with the known increase in amorphous character and reduced chain packing in CMC. Thus, the combined TGA, XRD, and ATR-FTIR data provide a coherent picture of structural disruption, decreased crystallinity, and weakened hydrogen bonding in THR-CMC compared with native cellulose. Thus, the combined TGA, XRD, and ATR-FTIR data provide a coherent picture of structural disruption, decreased crystallinity, and weakened hydrogen bonding in THR-CMC compared with native cellulose, explaining the observed mechanical and barrier properties of the composite films.

### 3.4. Characterization of Newly Developed Bioplastic Films

#### 3.4.1. Structural Characterization of Films

**Figure 3:**
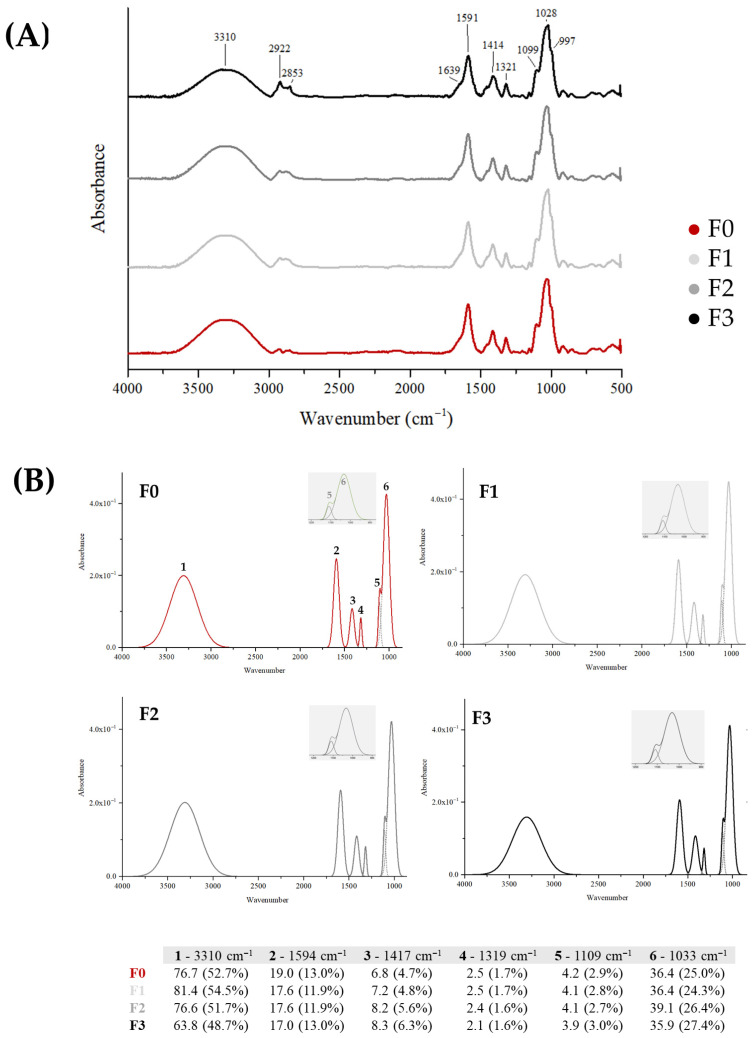
ATR-FTIR spectra (**A**) and deconvoluted peak profiles (**B**) of CMC-based films prepared from tomato processing residues and enriched with increasing doses of a polyphenolic extract (F0: control, no extract; F1: extract 40; F2: extract 60; F3: extract 100). Deconvolution analysis was carried out by fitting Gaussian functions to resolve overlapping peaks, allowing for the quantitative evaluation of the main spectral components and their relative contributions across the different film formulations.

ATR-FTIR spectra of four CMC-based films prepared from tomato processing residues and enriched with a polyphenolic extract at increasing doses are shown in Figure 3. The samples, labeled as F0 (control, no extract), F1 (extract 40 mg/100 mL), F2 (extract 60 mg/100 mL), and F3 (extract 100 mg/100 mL), exhibit the typical CMC absorption features.

The broad band around 3310 cm^−1^ corresponds to O–H stretching vibrations associated with hydroxyl groups and hydrogen bonding within the CMC and polyphenolic structures, while the peaks at 2922 and 2853 cm^−1^ are attributed to aliphatic C–H stretching from the polysaccharide backbone. The region between 1639 and 1591 cm^−1^ is mainly related to C=O and aromatic C=C stretching vibrations, whose intensity slightly increases with the polyphenolic extract content, indicating the incorporation of aromatic compounds. The bands at 1414 and 1321 cm^−1^ are ascribed to CH deformation and other vibrations typical of polysaccharide and phenolic groups, whereas the strong absorptions at 1099 and 1028 cm^−1^ correspond to C–O–C and C–O stretching of glycosidic and ether linkages, reflecting interactions between the CMC matrix and the incorporated phenolic species. The small peak near 997 cm^−1^ may arise from out-of-plane vibrations of aromatic substituents or carbohydrate structures. The ATR-FTIR deconvolution analysis provided further quantitative insight into these structural changes. The O–H stretching band at 3310 cm^−1^, associated with hydrogen-bonded hydroxyl groups, showed a slight increase in relative area from 52.7% in F0 to 54.5% in F1, followed by a decrease to 48.7% in F3. This trend suggests an initial enhancement of hydrogen bonding due to phenolic interactions, followed by a partial reduction as the phenolic content increased, likely due to structural rearrangements occurring in the later fractions. In contrast, the relative contribution of the band at 1417 cm^−1^ gradually increased from 4.7% in F0 to 6.3% in F3, indicating a progressive enrichment in carboxyl-containing components. The observed spectral shifts and redistribution of band areas suggest the establishment of new hydrogen bonds and associative interactions between CMC chains and phenolic structures, leading to a gradual structural rearrangement and enhanced molecular cohesion within the polymeric film network.

**Figure 4:**
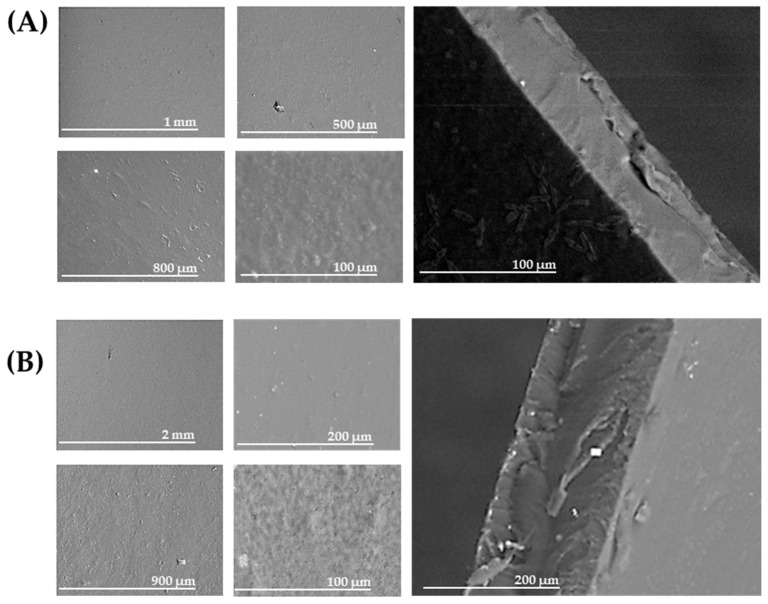
SEM micrographs of bio-based films obtained from (**A**) THR-cellulose and (**B**) THR-cellulose incorporated with the polyphenolic extract. Each panel presents images at variable magnifications chosen to best highlight relevant features: surface morphology is shown in the smaller-scale images, while the images depicting the film edges correspond to cross-section morphology. The addition of the extract slightly modified the surface texture, suggesting interactions between cellulose fibers and phenolic compounds. Differences in magnification reflect the need to capture distinctive structural details specific to each sample.

Figure 4 shows SEM micrographs of the neat CMC film F0 (A) and the film containing the tomato polyphenolic extract (F2; 60 mg/100 mL) (B). The polyphenol-enriched film exhibited a slightly rougher and more heterogeneous surface compared with the smooth morphology of neat CMC. At higher magnification, the TPPf film displayed small aggregates and micro-depressions across the surface, suggesting localized phase separation or rearrangement of the CMC network. Cross-sectional images confirmed that, while both films were cohesive, the extract-loaded sample exhibited a slightly less compact internal morphology with minor voids, likely arising from polymer-polyphenol interactions.

The structural and morphological insights obtained from ATR-FTIR and SEM analyses provide a rationale for the thermal behavior observed in the TGA/DTG experiments. In particular, the formation of additional hydrogen bonds and associative interactions between CMC chains and polyphenols, as well as the slightly rougher and more heterogeneous internal structure of the films, contribute to a more gradual and multistep thermal degradation profile. These interactions enhance molecular cohesion, delay the onset of major degradation events, and lead to a higher thermal stability of the polymer-polyphenol network, as reflected in the TGA/DTG thermograms. In fact, as depicted in Figure S9, the TGA/DTG thermogram of the F2 film shows a multistep thermal degradation behavior, which is typical of complex biopolymeric systems. Indeed, in the low-temperature region, approximately between 30 and 120 °C, a first moderate weight loss of about 10–15% is observed, accompanied by a small DTG signal. This stage could be mainly attributed to the evaporation of physically adsorbed and hydrogen-bonded water associated with the highly hydrophilic nature of CMC, as well as to the possible release of low-molecular-weight compounds from the polyphenolic extract. Between 120 and 220 °C, the weight decreases gradually, and only weak DTG features are detected. This region is associated with structural dehydration of CMC, the onset of thermal degradation of the less thermally stable polyphenolic compounds, and possible melting and molecular reorganization of carnauba wax, which may contribute to a certain stabilization of the system. The main degradation step occurs in the temperature range of approximately 220–320 °C and is characterized by a pronounced weight loss of about 40–45% and by two well-defined DTG peaks, indicating overlapping degradation mechanisms. This stage could be mainly related to depolymerization and backbone scission of the CMC chains, thermal degradation of the organic fraction of the polyphenolic extract, and partial decomposition of carnauba wax. The presence of multiple DTG peaks suggests interactions among the biofilm components, leading to non-simultaneous degradation processes. At the highest temperature range, up to 600 °C, the weight loss becomes gradual, and the DTG signal approaches zero. This region corresponds to the carbonization and slow degradation of the most thermally stable residues, resulting in a final solid residue of approximately 28–30%, which can be attributed to thermally resistant components.

The combined characterization of the films by ATR-FTIR, SEM, and TGA/DTG provides a comprehensive understanding of their structural and functional properties. ATR-FTIR analysis revealed the incorporation of polyphenols into the CMC matrix, highlighting the formation of new hydrogen bonds and associative interactions. SEM imaging confirmed these interactions by showing a slightly rougher surface and more heterogeneous internal morphology in extract-loaded films, suggesting localized phase separation and polymer–polyphenol rearrangements. These microstructural modifications are further supported by TGA/DTG results, which demonstrate enhanced thermal stability and multistep degradation behavior, indicative of strong polymer-polyphenol interactions.

#### 3.4.2. Physical Properties of Films

Different physical properties, i.e., thickness, chroma measurements (transparency, whiteness, and yellowness index), moisture content, and water vapor transmission rate (*WVTR*) of newly developed films are summarized in Table 2.

All the films exhibited 0.11 ± 0.01 mm thickness. The moisture content (%) of films was in the range of 6.93 ± 1.97% (F2) and 10.37 ± 0.77 (F0), indicating a slight reduction by the addition of TPPf. Moreover, the addition of TPPf resulted in a notable change in the chroma properties of films. The control film (F0; without extract) showed 84.12 ± 2.39 whiteness index and −10.10 ± 0.01 yellowness index, reflecting a bluish hue (not yellowish). The addition of TPPf progressively reduced the whiteness index and enhanced the yellowness index. Film F1 (40 mg/100 mL) showed 77.48 ± 1.11 whiteness index and 22.76 ± 1.12 yellowness index, indicating a shift towards yellow hue by the addition of polyphenolic fractions. Similarly, F2 (60 mg/100 mL) and F3 (100 mg/100 mL) indicated 66.88 ± 1.05 and 44.85 ± 1.68 whiteness index and 46.28 ± 1.50 and 93.18 ± 3.49 yellowness index, respectively. This means that an increased TPPf content led to a more yellow appearance. The shift from transparent white to yellowish-toned hue could be attributed to the intrinsic coloration of polyphenols. Polyphenolic compounds are known to possess chromophoric structures that contribute to a yellowish/brownish appearance when incorporated into polymer matrices. Furthermore, slightly decreased transparency by the addition of TPPf was observed: from 2.83 ± 0.00 (F0) to 2.78 ± 0.00 (F1–F3, TPPf-containing films). A similar chroma shift is reported by Thivya et al. [46] who developed Na-alginate/CMC–based films incorporating shallot waste extract. Similarly, Rambabu & Bharath [47] developed chitosan films incorporating mango leaf extract. These results reveal an increased light barrier ability of polyphenol-enriched films useful for ‘active’ food packaging.

The films were evaluated for their water vapor transmission rate (*WVTR*) after 24 h (*WVTR*_24_) and 48 h (*WVTR*_48_), as described in Table 2. The film without TPPf (F0) showed *WVTR* values of 7.28 ± 0.13 g/m^2^ × h (24 h), and 4.22 ± 07 g/m^2^ × h (48 h). Films F1 (40 mg/100 mL) and F2 (60 mg/100 mL) showed a significant (*p* < 0.05) reduction in *WVTR*. This improvement in barrier properties was likely due to enhanced intermolecular hydrogen bonding and compact polymer network between THR-CMC and polyphenolic compounds, which reduces water vapor diffusion. These results are well aligned with previous findings reporting reduction in water vapor permeability of pectin films incorporated with green tea extract [48]. The extract served as a tightening agent in film matrix and raising crystallinity. Similar findings were reported by Szymański et al. [49] by incorporating herbal (*Rumex hydrolapathum*) extract into pectin/curdlan blended films. The extract incorporation reduced the *WVTR* due to increased hydrophobic interactions that lengthened water diffusion pathways. Conversely, at the highest TPPf dose level (F3), *WVTR* increased sharply. This behavior is consistent with previous studies indicating that high loadings of hygroscopic polyphenols enhance the hydrophilicity of the film matrix, promoting water vapor transmission [50]. In a nutshell, the data reflect biphasic behavior: moderate TPPf loading improves water barrier properties, whereas higher loadings decrease the barrier, likely due to increased hydrophilicity and partial disruption of the polymer network.

#### 3.4.3. Mechanical Properties of Films

The mechanical properties of films are critical parameters for understanding their suitability for food packaging to maintain integrity during handling, transportation, and storage [3].

**Figure 5:**
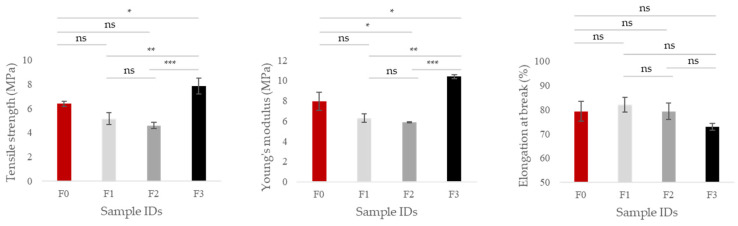
Mechanical properties: tensile strength (MPa), Young’s modulus (MPa), and elongation at break (%) of newly developed THR-CMC-based films without/with different concentrations of TPPf. Statistical significance was determined by one-way ANOVA followed by Tukey’s post hoc test. Differences were considered significant at *p* < 0.05 (*), *p* < 0.01 (**), and *p* < 0.001 (***); ns = not significant.

The mechanical parameters of THR-CMC-based films without and with different concentrations of TPPf are presented in Figure 5. The tensile strength and Young’s modulus of films showed a non-monotonic trend depending on the TPPf concentration, reflecting the dual effect of the polyphenolic fraction on the polymer matrix. The control film (F0) exhibited a comparatively higher tensile strength (6.39 ± 0.21 MPa) and Young’s modulus (7.96 ± 0.90 MPa) than both F1 (40 mg/100 mL) and F2 (60 mg/100 mL). Film F1 displayed a tensile strength of 5.16 ± 0.51 MPa and Young’s modulus of 6.29 ± 0.40 MPa, while F2 exhibited even less tensile strength (4.60 ± 0.25 MPa) and Young’s modulus (5.86 ± 0.05 MPa) than F0 and F1. This initial decrease in stiffness can be attributed to the plasticizing effect of the polyphenolic fraction, which increases polymer chain mobility at low to moderate loadings [51,52]. Film F3 (100 mg/100 mL) showed significantly (*p* < 0.05) increased tensile strength (7.85 ± 0.64 MPa) and Young’s modulus (10.39 ± 0.18 MPa) partially recovering towards the F0 or even exceeding. However, the elongation at break value was slightly reduced from 79.34 ± 4.15% (F0) to 72.96 ± 1.35 (F3), but this change was not statistically significant (*p* > 0.05). These results confirm that TPPf exerts a biphasic effect on film properties: at low concentrations it acts as a plasticizer, reducing stiffness, whereas at high concentrations it forms reinforcing aggregates, increasing tensile strength and modulus. In a similar study, Nastasi et al. [53] reported that polyphenol-rich fruit extract improves the mechanical properties of pectin films. Indeed, the tensile strength of F0 film is in good agreement with literature values for commercial CMC films, such as those reported by Jannatyha et al. [54], who found 6.10 MPa. This confirms that the baseline mechanical performance of our films is consistent with established data and provides a reliable reference for evaluating the effect of TPPf addition on film properties. It should be noted that the mechanical performance of CMC films reported in the literature varies widely depending on formulation and processing parameters. For example, tensile strength values as high as 27.5 MPa have been reported for CMC-based films plasticized with glycerol. In another study, Klunklin et al. [55] reported a tensile strength of 44.59 MPa for CMC films (DS = 0.98) derived from asparagus cellulose. These differences can be attributed to variations in molecular weight, degree of substitution, plasticizer content, relative humidity during testing, and film preparation conditions. Therefore, direct numerical comparison should be made with caution. Interestingly, the trends observed for *WVTR*, and mechanical properties reflect complementary aspects of film structure. At low TPPf concentrations (F1 and F2), the extract enhances polymer chain mobility and compactness, improving water barrier properties while slightly reducing stiffness due to a plasticizing effect. At the highest concentration (F3), TPPf molecules aggregate to form hydrogen-bonded clusters, increasing stiffness and tensile strength, while the hygroscopic nature of the high polyphenol content increases water affinity, resulting in a higher *WVTR*. Thus, the apparent discrepancy between barrier and mechanical properties is a consequence of the dual role of TPPf: modifying water transport pathways through hydrophilicity and simultaneously reinforcing the polymer network through aggregation. These observations, together with the intrinsic water solubility of THR-CMC-based films due to the presence of carboxymethyl groups and glycerol, limit their potential applications to dry food packaging or surface coatings for low-moisture foods.

#### 3.4.4. UV-Blocking and Antiradical Activity of Films

The UV-blocking properties of the CMC-based films were evaluated by recording their transmittance spectra in the 200–800 nm range using a UV-Vis spectrophotometer.

**Figure 6:**
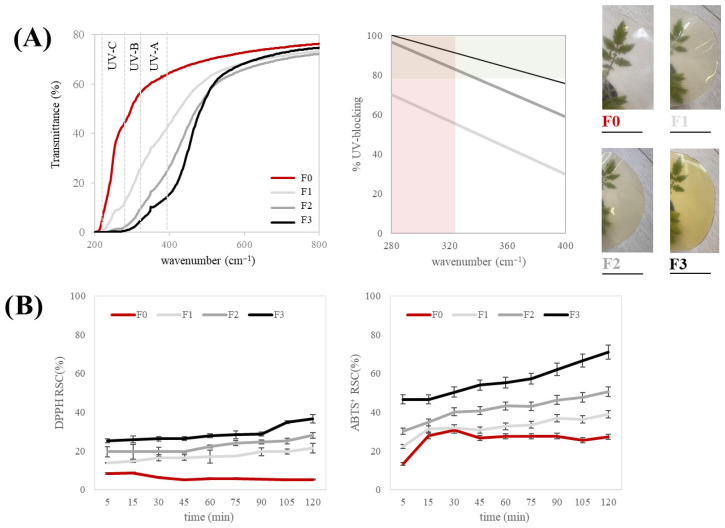
Optical and antioxidant properties of CMC-based films enriched with different concentrations of the tomato polyphenolic fraction (TPPf): F0 (control, no extract), F1 (40 mg/100 mL), F2 (60 mg/100 mL), and F3 (100 mg/100 mL). (**A**) Visual appearance of the films, showing a gradual increase in color intensity with extract addition, and UV-blocking ability of the films. (**B**) DPPH radical scavenging capacity (RSC%) and ABTS^+^ radical scavenging capacity (RSC%) of the films at different reaction times. Data are expressed as mean ± SD (*n* = 3).

Sunlight ultraviolet (UV) radiation comprises UV-A (320–400 nm), UV-B (280–320 nm), and UV-C (220–280 nm) wavelengths [22]. The control film (F0) exhibited high transmittance in the 200–400 nm region, revealing the poor intrinsic UV-shielding ability of the neat CMC matrix. In contrast, the films enriched with the tomato polyphenolic fraction (TPPf) showed significantly (*p* < 0.05) lower transmittance, particularly within the UV-A (320–400 nm) and UV-B (280–315 nm) regions (Figure 6A). This effect became more pronounced as the extract concentration increased from F1 (40 mg/100 mL) to F3 (100 mg/100 mL). Film F3 showed the most effective UV protection, with nearly zero transmittance below 280 nm, corresponding to the UV-C range. The enhanced UV-blocking capacity is due to phenolic compounds with conjugated aromatic rings and hydroxyl groups that absorb UV photons and convert them into less energetic forms. These features make polyphenols effective natural UV filters. Similar behavior has been observed in biopolymer films containing plant-derived antioxidants, where phenolic conjugation improves light absorption and protects the material from photodegradation [56]. To better contextualize the UV-blocking performance, comparison with commercially available and literature-reported packaging materials is informative. Conventional petroleum-based polymers widely used in food packaging, such as polyethylene (PE) and polypropylene (PP), generally exhibit limited intrinsic UV-blocking capacity due to their saturated aliphatic backbone, which lacks chromophoric groups capable of absorbing UV radiation. For example, high-density PE films commonly used in agricultural and packaging applications show high UV transmittance in the 200–400 nm range, indicating low UV attenuation [57]. Similarly, PP films are prone to photodegradation under UV exposure in the absence of stabilizers, confirming their poor inherent UV protection [58]. Poly(ethylene terephthalate) (PET), owing to its aromatic backbone, demonstrates improved attenuation of short-wavelength UV radiation compared with PE and PP; however, PET films still do not fully block UV-A and UV-B wavelengths without specific UV absorbers or multilayer barrier structures [59].

Several bio-based and hybrid UV-shielding films have been developed to overcome these limitations. Poly(vinyl alcohol) (PVA) composites with wood nanofibers enhance UV shielding through increased scattering and absorption [60]. Plant-derived phenolic compounds have been shown to provide nearly complete UV-B blocking; for instance, the addition of naringin to cellulose films resulted in UV-B blocking efficiencies approaching 100% at 5% and 20% naringin loading, while maintaining high visible transparency (~85% at 550 nm) [61]. Other natural phenolics such as ellagic acid, curcumin, and tannic acid also enhance UV shielding through hydrogen bonding or supramolecular structuring, with reported UV-blocking efficiencies reaching 99% [62]. Synthetic additives can also provide strong UV protection; for example, incorporation of benzoxazine into poly(lactic acid) (PLA) resulted in a UV-blocking efficiency of 98.3% at 350 nm compared with 6.7% for pristine PLA [62]. Bio-based polyesters such as poly(butylene adipate-co-terephthalate) (PBAT) or PLA films containing plant extracts or phenolic antioxidants have achieved UV-blocking efficiencies above 80–90% in the UV-A and UV-B regions [63].

In this context, the CMC-TPPf films exhibited average UV-blocking efficiencies ranging from 42.10% to 83.32% in the UV-B region and from 64.14% to 96.32% in the UV-A region, depending on extract concentration. In particular, F3 achieved UV-A and UV-B blocking values comparable to or approaching those of other bio-based antioxidant-enriched systems, without the need for inorganic nanoparticles or synthetic UV absorbers. While commercial PE and PP films allow high UV transmittance and PET only partially attenuates UV-A/B without stabilizers, the CMC/TPPf films achieve UV-blocking efficiencies approaching or exceeding 80–90%, comparable to bio-based antioxidant-enriched systems reported in the literature. These findings highlight the effectiveness of tomato-derived polyphenols as natural, food-compatible UV-shielding agents and support the potential application of CMC/TPPf films in light-sensitive food packaging.

The antioxidant activity of the films was assessed through DPPH and ABTS radical scavenging assays (Figure 6B). Both tests showed a clear time-dependent increase in radical scavenging capacity (RSC%), although the films were far less effective than the extract alone, which was able to completely quench the radicals at 250 µg/mL. This behavior reflects the gradual release of phenolic compounds from the polymer matrix and their interaction with radicals in solution. The F0 film showed limited antioxidant performance, consistent with the low reactivity of pure CMC. In contrast, the incorporation of TPPf markedly enhanced radical scavenging efficiency. In the DPPH assay, RSC% increased with the concentration of extract, reaching approximately 24% for F3 after 90 min, compared with 18% for F2, 14% for F1, and 8% for F0. A similar trend was observed in the ABTS assay, where F3 again displayed the highest antioxidant capacity, followed by F2 and F1. The improved antioxidant activity can be attributed to the flavonoids and hydroxycinnamic acid derivatives identified in the TPPf, particularly rutin and chlorogenic compounds, which are known to act as efficient hydrogen or electron donors.

#### 3.4.5. Mechanistic Interpretation of Film Behavior

The incorporation of TPPf into the THR-CMC matrix induced a series of structural and functional modifications that can be attributed to specific molecular interactions between the polyphenolic compounds and the polysaccharide chains. ATR-FTIR analysis revealed an increase in the intensity of the O–H stretching band and variations in the hydrogen bonding index (HBI), indicating enhanced intra- and intermolecular hydrogen bonding. Polyphenols are able to form multiple hydrogen bonds with the hydroxyl and carboxylate groups of CMC, acting as physical crosslinking points and promoting a more compact and stabilized network. Similar hydrogen-bond-mediated interactions between flavonoids and CMC have been reported in CH-Fla-CMC composites [64]. Likewise, the adsorption of phenolic acids such as caffeic, chlorogenic, and gallic acid onto cellulose matrices has been shown to significantly modify film structure and mechanical behavior through analogous bonding mechanisms [65]. These interactions are consistent with the broader structural role of polyphenols in polymer networks, where hydrogen bonding and π–π interactions contribute to network reinforcement and functional enhancement [66]. This structural reinforcement explains the observed increase in tensile strength and Young’s modulus in the polyphenol-enriched films. The additional hydrogen bonding reduces the segmental mobility of the polymer chains, leading to a more rigid and ordered matrix. Conversely, the decrease in elongation at break reflects the reduced ability of the material to undergo plastic deformation, a typical behavior of polysaccharide films with high intermolecular cohesion. Similar correlations between polyphenol-induced network densification and mechanical strengthening have been reported in cellulose-polyphenol films derived from apple processing waste [7] and in other biopolymer systems enriched with phenolic compounds [22]. SEM micrographs further support this interpretation. Films containing TPPf exhibited smoother surfaces and more homogeneous cross-sections compared to the control, suggesting good compatibility between CMC and the polyphenolic fraction. The absence of phase separation or aggregated domains indicates that the interactions between the two components are sufficient to maintain a continuous and cohesive structure, consistent with previous observations in cellulose–polyphenol systems [7,66]. The more compact morphology observed in the enriched films aligns with the mechanical reinforcement and reduced permeability, as a denser microstructure typically limits water vapor diffusion pathways [22].

Barrier properties were also influenced by the structural reorganization induced by TPPf. At low concentrations, the denser polymer network reduced water vapor transmission by restricting diffusion channels for water molecules. At higher concentrations, however, the excess of hydrophilic phenolic groups or the formation of micro-irregularities may increase moisture affinity, resulting in a slight rise in *WVTR*. Similar concentration-dependent trends have been reported in biopolymer films enriched with plant-derived phenolic extracts [7,22].

Finally, the strong UV-blocking ability of the enriched films is directly related to the presence of conjugated aromatic structures typical of polyphenols, which absorb efficiently in the 250–350 nm region. The homogeneous dispersion of TPPf within the CMC matrix enables the development of films with intrinsic UV-shielding properties without the need for synthetic additives, in agreement with previous studies on cellulose-polyphenol composites [7].

### 3.5. Biodesintegration of the Obtained Films

**Figure 7:**
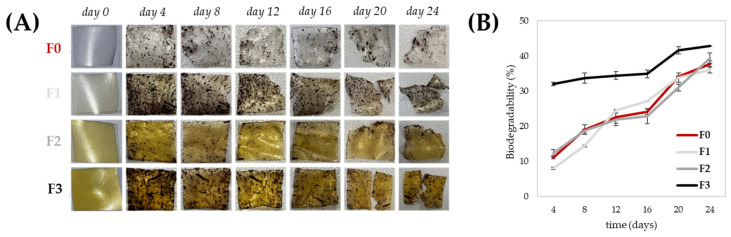
Biodesintegration of THR-CMC-based films enriched with different concentrations of the tomato polyphenolic fraction (TPPf): F0 (control, no extract), F1 (40 mg/100 mL), F2 (60 mg/100 mL), and F3 (100 mg/100 mL). (**A**) Representative images of the samples subjected to the soil burial test at different time points; (**B**) weight loss (%) measured over 24 days. Samples were collected every four days, cleaned, and reweighed. Data are expressed as mean ± SD (*n* = 3).

Biodesintegration of the CMC-based films was evaluated via a 24-day soil burial biodesintegration test, with samples retrieved every four days, cleaned, and weighed to assess weight loss (Figure 7).

All films showed significant degradation, exceeding 35% after 24 days. Low concentrations of tomato polyphenolic fraction (TPPf) in films F1 and F2 did not notably affect degradation compared to F0, while the highest concentration in F3 accelerated weight loss. These results indicate that increasing polyphenol levels not only enhances antioxidant and UV-blocking properties but also influences the degradation behavior of the films, highlighting that different functional performances can be obtained within the same cellulose-based matrix by modulating the TPPf content. This enhanced biodegradation is likely due to phenolic–polymer interactions that partially disrupt the polymer network, increasing water uptake and microbial accessibility. Similar effects have been observed in other biopolymer films enriched with plant polyphenols, where natural antioxidants promote microbial colonization and enzymatic degradation through increased hydrophilicity and micro-porosity [67]. Taken together, these results demonstrate that tomato processing residues provide a highly versatile cellulose-based matrix, whose functional and physicochemical properties can be tailored through the incorporation of polyphenols, enabling diverse applications while maintaining the sustainability of the upcycled system. From a broader resource-efficiency perspective, this behavior further supports the sustainability of the proposed system, which enables the simultaneous recovery and utilization of both structural polysaccharides and bioactive compounds from tomato processing residues. Starting from 100 g of dry THR residue (post-SFE), 39.7 g of crude ethanolic extract were obtained, yielding 4.6 g of purified polyphenolic fraction, while cellulose extraction provided a 22% recovery subsequently converted into water-soluble CMC. At larger scale, this corresponds to approximately 46 kg of polyphenolic extract and 220 kg of cellulose per metric ton of dry biomass. Such integrated recovery enhances the overall valorization efficiency of THR and reinforces the industrial relevance of this cascade biorefinery strategy within a circular economy framework.

## 4. Conclusions

This study demonstrates that the polyphenolic fraction extracted from exhausted tomato peels (TPPf) can be successfully incorporated into carboxymethyl cellulose films derived from the same tomato supply chain, yielding biodegradable materials with enhanced UV protection, antioxidant activity, and overall biodegradability. Structural analyses confirmed strong polymer–phenolic compatibility with only minor microstructural changes, indicating that film integrity is maintained. The results highlight a circular innovation model, in which tomato processing by-products are valorized as bioactive compounds and reintegrated into functional biopolymers, linking sustainability, technological innovation, and materials science. Notably, while F3 (100 mg/100 mL TPPf) exhibits the highest tensile strength and Young’s modulus due to the formation of hydrogen-bonded clusters, it also shows an increased water vapor transmission rate compared with F1 and F2, likely reflecting the hygroscopic nature of high TPPf content. This trade-off underscores that the optimal formulation depends on the intended application: F3 is preferable for maximum mechanical reinforcement, whereas F1 or F2 may be more suitable when barrier properties are prioritized. Finally, to ensure industrial feasibility and sustainable scale-up, energy-intensive steps in extraction and film production should be optimized or replaced with less energy-demanding alternatives. Thus, the study demonstrates how functional materials with tailored properties can be obtained from food waste while advancing circular and low-impact agri-food systems.

## Figures and Tables

**Table 1:** Tentative identification of compounds in the tomato polyphenolic fraction (TPPf) based on TOF-MS and TOF-MS/MS data. RDB = Ring and Double Bond equivalents.

Peak	tR		Tentative Assignment	Formula	[M − H]^−^ (*m*/*z*)	RDB	Error (ppm)
1	1.265	*	3-CQA	C_16_H_18_O_9_	353.0870	8	−2.3
2	1.457	*	Caffeic acid hexoside 1	C_15_H_18_O_9_	341.0869	7	−2.7
3	1.767	*	Caffeic acid hexoside 2	C_15_H_18_O_9_	341.0872	7	−1.8
4	1.890	*	Dihydrocaffeic acid hexoside 1	C_15_H_20_O_9_	343.1018	6	−4.8
5	2.128	*	Dihydrocaffeic acid hexoside 2	C_15_H_20_O_9_	343.1031	6	−1.0
6	2.444	*	Caffeic acid hexoside 3	C_15_H_18_O_9_	341.0873	7	−1.5
7	2.794	*	4-CQA	C_16_H_18_O_9_	353.0870	8	−2.3
8	3.118	†	Tuberonic acid hexoside 1	C_18_H_28_O_9_	387.1652	5	−2.2
9	3.308	‡	Benzyl pentosyl hexoside	C_18_H_26_O_10_	401.1454	6	0.2
10	3.600	*	5-CQA	C_16_H_18_O_9_	353.0862	8	−4.5
11	3.705	‡	Hydroxyphenethyl deoxyhexosylhexoside	C_20_H_30_O_11_	445.1709	6	−1.4
12	4.002	†	Tuberonic acid hexoside 2	C_18_H_28_O_9_	387.1653	5	−2.0
13	4.587	**	Quercetin dihexosyl deoxyhexoside	C_33_H_40_O_21_	771.1998	14	1.1
14	4.826	‡	Phenethyl pentosyl hexoside	C_19_H_28_O_10_	415.1604	6	−1.4
15	6.558	**	Eriodictyol 7-*O*-hexoside	C_21_H_22_O_11_	449.1084	11	−1.2
16	6.724	**	Naringenin 7-*O*-hexoside	C_21_H_22_O_10_	433.1128	11	−2.8
17	7.091	⁑	*p*-Coumaroyltyramine hexoside	C_23_H_27_NO_8_	444.1656	11	−1.8
18	7.166	**	Quercetin pentosyl rutinoside	C_32_H_38_O_20_	741.1877	14	−0.9
19	7.537	**	Quercetin deoxyhexosylhexoside	C_27_H_30_O_16_	609.1454	13	−1.2
20	7.757	**	Phloretin 3’,5’-di-*C*-glucoside	C_27_H_34_O_15_	597.1821	11	−0.7
21	8.014	**	Kaempferol pentosyl rutinoside	C_32_H_38_O_19_	725.1910	14	−3.4
22	8.033	**	Naringenin *O*-hexoside	C_21_H_22_O_10_	433.1129	11	−2.6
23	8.271	*	3,4-diCQA	C_25_H_24_O_12_	515.1187	14	−1.6
24	8.601	*	3,5-diCQA	C_25_H_24_O_12_	515.1186	14	−1.7
25	8.676	**	Kaempferol rutinoside	C_27_H_30_O_15_	593.1494	13	−3.0
26	8.764	†	caffeoylhexosyl tuberonic acid	C_27_H_34_O_12_	549.1972	11	−1.0
27	8.975	*	Dicaffeoyl hexose	C_24_H_24_O_12_	503.1188	13	−1.4
28	9.560	*	4,5-diCQA	C_25_H_24_O_12_	515.1183	14	−2.3
29	9.736	**	Naringenin chalcone *O*-hexoside	C_21_H_22_O_10_	433.1134	11	−1.4
30	10.536	**	Quercetin pentosyl coumaroyl rutinoside	C_41_H_44_O_22_	887.2208	20	−4.9
31	12.056	**	Naringenin	C_15_H_12_O_5_	271.0599	10	−4.8
32	12.586	**	Hesperitin	C_16_H_14_O_6_	301.0713	10	−1.5
33	13.069	**	Naringenin chalcone	C_15_H_12_O_5_	271.0612	10	0
34	13.202	*	TriCQA	C_34_H_30_O_15_	677.1486	20	−3.8

* Hydroxycinnamoyl compounds; ** Flavonoids; † Tuberonic acid glycosides; ‡ Aromatic glycosides; ⁑ Phenylamide.

**Table 2:** Physical properties of newly developed films without/with different concentrations of TPPf. Statistical significance was determined by one-way ANOVA followed by Tukey’s post hoc test. Differences were considered significant at * *p* < 0.05, ** *p* < 0.01, *** *p* < 0.001, **** *p* < 0.0001, NS: not significant.

**Sample IDs**	**Film Thickness (mm)**	**Color Analysis**		**Transparency Index**	**Moisture Content (%)**	**Water Vapor Transmission Rate (g/m^2^ × h)**	
		**Whiteness Index**	**Yellowness Index**			***WVTR*** **_24_**	***WVTR*** **_48_**
F0	0.11 ± 0.01	84.12 ± 2.39	−10.10 ± 0.01	2.83 ± 0.00	10.37 ± 0.77	7.28 ± 0.13	4.22 ± 0.07
F1	0.11 ± 0.01 ^NS^	77.48 ± 1.11 ****	22.76 ± 1.12 ****	2.78 ± 0.00 ^NS^	8.27 ± 1.05 ^NS^	2.10 ± 0.44 ****	1.30 ± 0.13 *
F2	0.11 ± 0.01 ^NS^	66.88 ± 1.05 ****	46.28 ± 1.50 ****	2.78 ± 0.00 ^NS^	6.93 ± 1.97 **	2.65 ± 0.16 ****	2.24 ± 0.06 ^NS^
F3	0.11 ± 0.01 ^NS^	44.85 ± 1.68 ****	93.18 ± 3.49 ****	2.78 ± 0.00 ^NS^	7.09 ± 0.19 **	16.31 ± 0.06 ****	8.42 ± 0.41 ***

## Data Availability

The original contributions presented in this study are included in the article/Supplementary Material. Further inquiries can be directed to the corresponding author.

## Supplementary Materials

The following supporting information can be downloaded at: https://www.mdpi.com/article/10.3390/foods15061067/s1, Figure S1: TOF-MS/MS spectra of glycosylated flavonols: (A) quercetin as aglycone; (B) kaempferol as aglycone; Figure S2: TOF-MS/MS spectra of naringenin and naringenin chalcone and their hexosides; Figure S3: TOF-MS/MS spectrum of phloretin 3’,5’-di-*C*-glucoside; Figure S4: TOF-MS/MS spectra of monocaffeoyl quinic acid isomers (1, 7, and 10) and dihydrocaffeoyl quinic acid isomers (23, 24, and 28), and tricaffeoyl quinic acid (34); Figure S5. TOF-MS/MS spectra of caffeoyl hexosides (2, 3, and 6) and dihydrocaffeoyl hexosides (4, and 5); Figure S6: TOF-MS/MS spectrum of the phenolamide *p*-coumaroyltyramine hexoside; Figure S7: TOF-MS/MS spectra of aromatic glycosides (9, 11, and 14); Figure S8. TOF-MS/MS spectra of tuberonic acid derivatives (8, 12, and 26); Figure S9: Thermogravimetric analysis (TGA) and derivative thermogravimetric (DTG) curves of the F2 film obtained from tomato residues.
