# Supplementary material for: Upcycling Tomato Harvest and Processing Residues into Polyphenol-Enriched Cellulosic Films with Tunable Antioxidant and UV-Blocking Properties
Source: Foods. 2026 Mar 18;15(6):1067. doi: 10.3390/foods15061067 (PMC13024965; doi:10.3390/foods15061067)
Supplement: Supplementary file 1 [file foods-15-01067-s001.zip › foods-4166172-supplementary.pdf]

Supplementary materials

Upcycling Tomato Harvest and Processing Residues into Polyphenol-Enriched Cellulosic Films with Tunable Antioxidant and UV-Blocking Properties

Sarmad Ahmad Qamar [1], Simona Piccolella [1], Raffaele Raimondo [2,†] and Severina Pacifico [1,*]

[1] Department of Environmental, Biological & Pharmaceutical Sciences and Technologies, University of Campania 'Luigi Vanvitelli', Via Vivaldi 43, 81100 Caserta, Italy; sarmadahmad.qamar@unicampania.it (S.A.Q.); simona.piccolella@unicampania.it (S.P.)

[2] Department of Science and Technology, Parthenope University of Naples, Centro Direzionale, Isola C4, 80143 Naples, Italy; raffaele.raimondo@uniparthenope.it or raffaeleraimondo@mater.it

* Correspondence: severina.pacifico@unicampania.it; Tel.: +39(0)823274578

† Current address: Mater S.R.L., Via Brecce a S. Erasmo, 114, 80146 Naples, Italy.

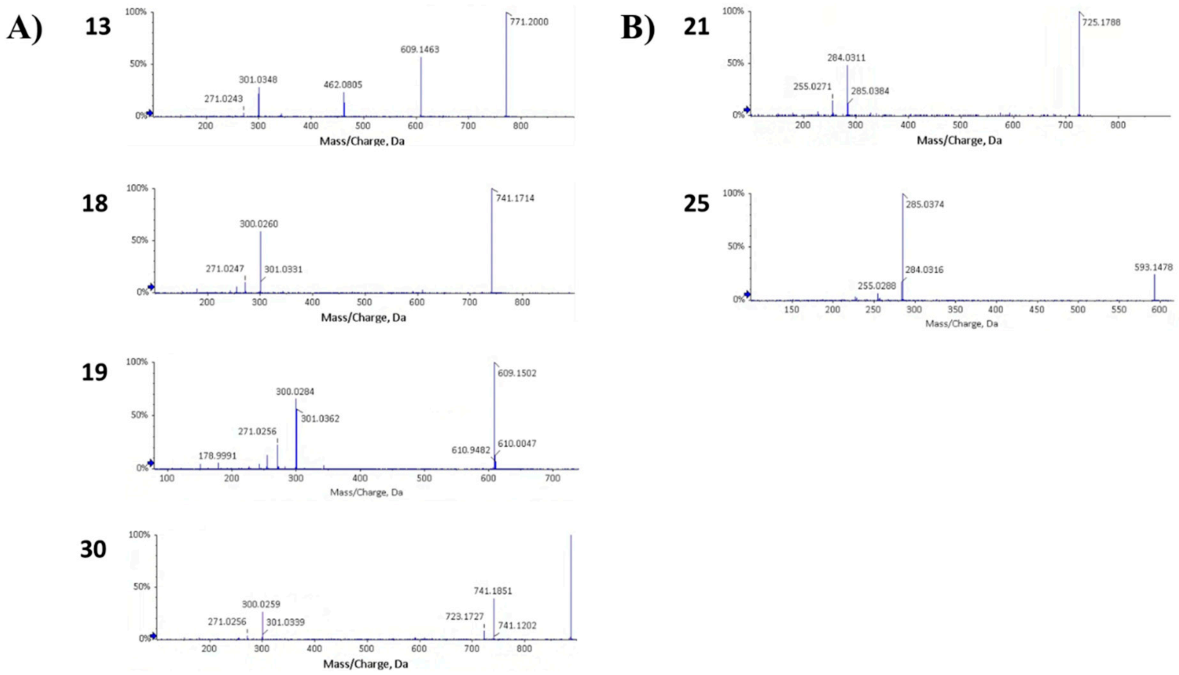

Figure S1. TOF-MS/MS spectra of glycosylated flavonols: A) quercetin as aglycone; B) kaempferol as aglycone.

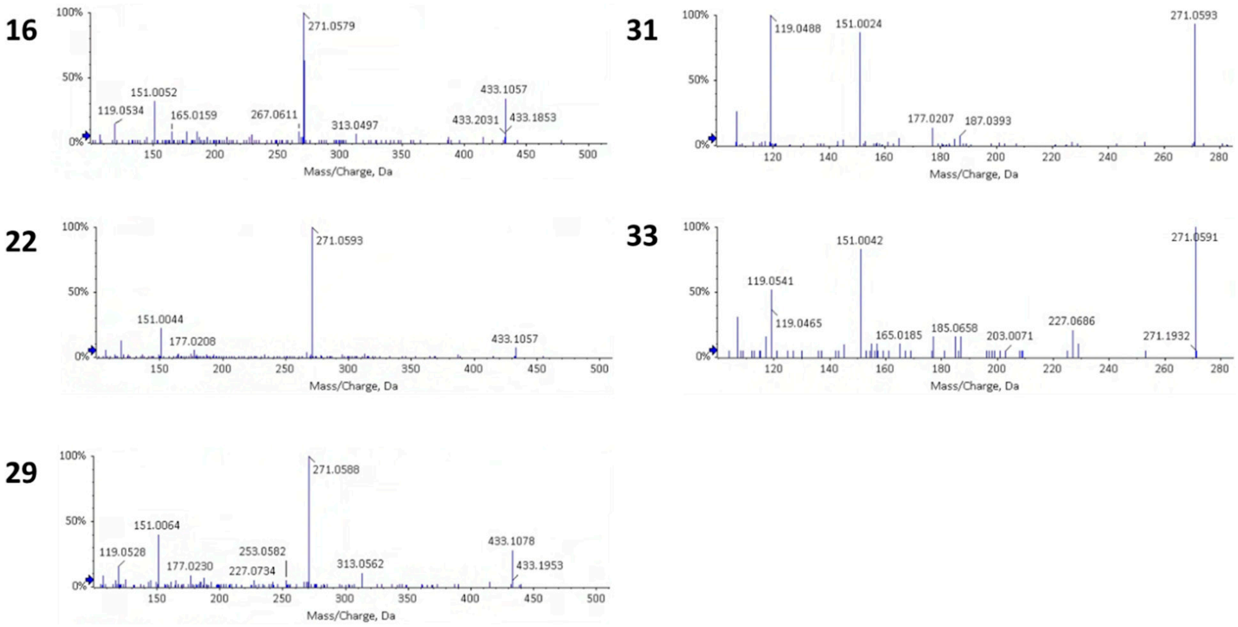

Figure S2. TOF-MS/MS spectra of naringenin and naringenin chalcone and their hexosides.

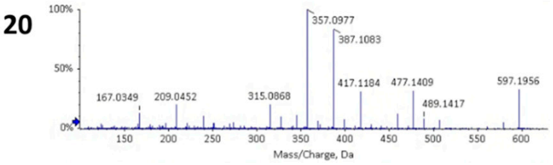

Figure S3. TOF-MS/MS spectrum of phloretin 3′,5′-di-C-glucoside.

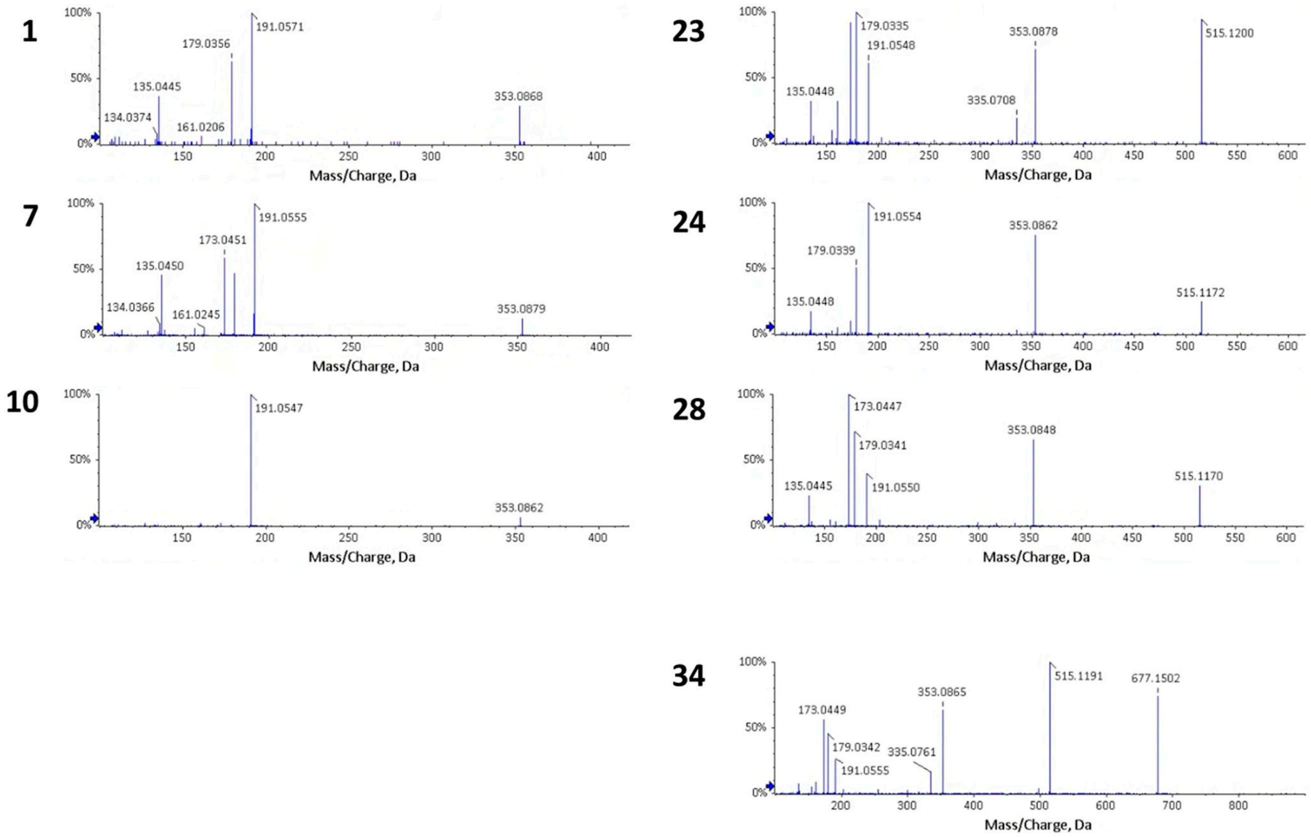

Figure S4. TOF-MS/MS spectra of monocaffeoyl quinic acid isomers (**1**, **7**, and **10**) and dihydrocaffeoyl quinic acid isomers (**23**, **24**, and **28**), and tricaffeoyl quinic acid (**34**).

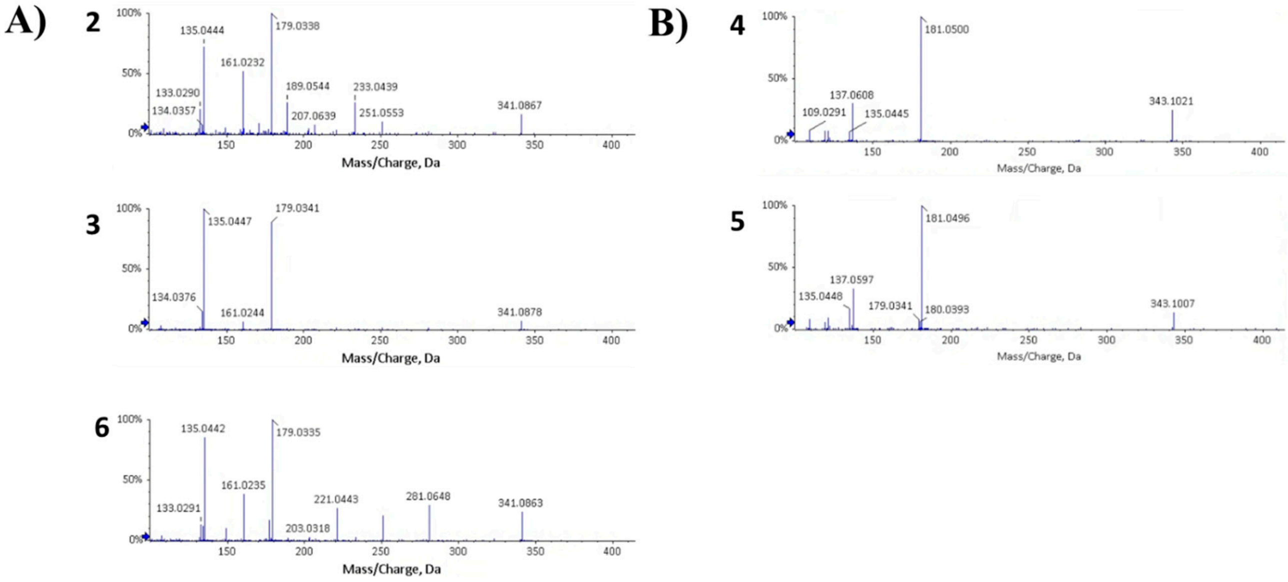

Figure S5. TOF-MS/MS spectra of caffeoyl hexosides (**2**, **3**, and **6**) and dihydrocaffeoyl hexosides (**4**, and **5**).

17

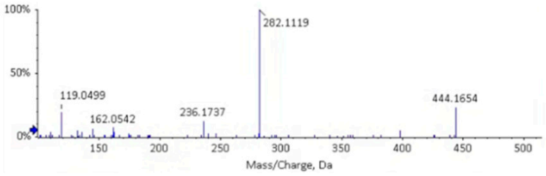

Figure S6. TOF-MS/MS spectrum of the phenolamide *p*-coumaroyltyramine hexoside.

9

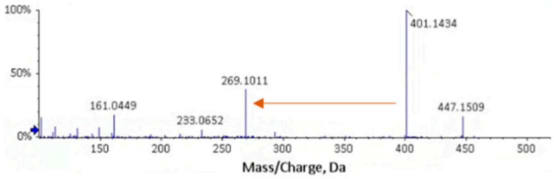

11

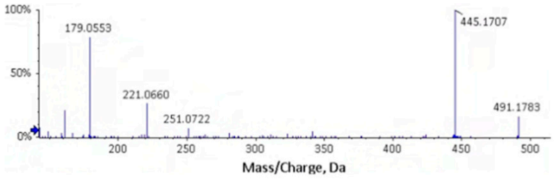

14

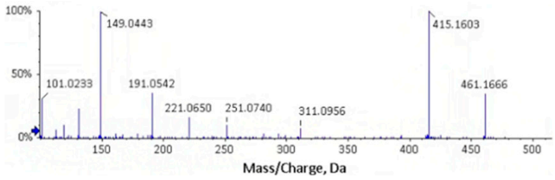

Figure S7. TOF-MS/MS spectra of aromatic glycosides (**9**, **11**, and **14**).

8

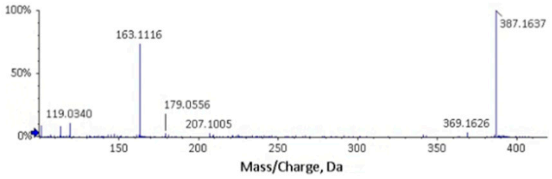

12

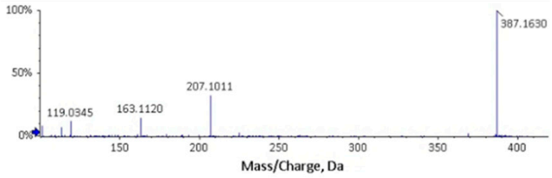

26

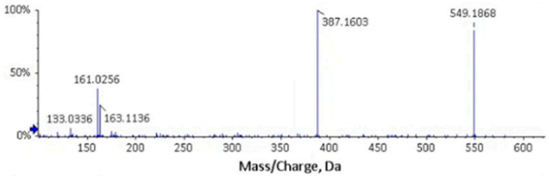

Figure S8. TOF-MS/MS spectra of tuberonic acid derivatives (**8**, **12**, and **26**).

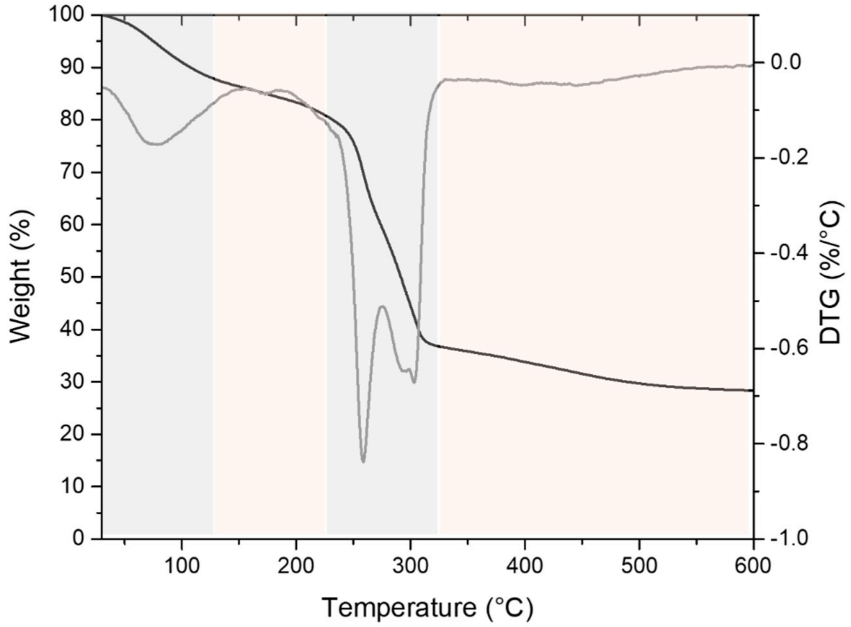

Figure S9. Thermogravimetric analysis (TGA) and derivative thermogravimetric (DTG) curves of the F2 film obtained from tomato residues.
